# Prodrug AST-003 Improves the Therapeutic Index of the Multi-Targeted Tyrosine Kinase Inhibitor Sunitinib

**DOI:** 10.1371/journal.pone.0141395

**Published:** 2015-10-29

**Authors:** Qiang Huang, Changhua Zhou, Xiao Chen, Bing Dong, Siqi Chen, Ning Zhang, Yawei Liu, Anrong Li, Meicun Yao, Ji Miao, Qing Li, Zhong Wang

**Affiliations:** 1 School of Pharmaceutical Sciences, Sun Yat-Sen University, Guangzhou, China; 2 Centre for Cellular & Structural Biology, Sun Yat-Sen University, Guangzhou, China; 3 Peking University Cancer Hospital, Beijing Institute for Cancer Research, Beijing, China; 4 Health Division of Guard Bureau, General Staff Department of PLA, Beijing, China; 5 Ascenta Pharmaceuticals, Guangzhou, Guangdong, P. R. China; University of Kentucky, UNITED STATES

## Abstract

Patients have responded well to the multi-targeted tyrosine kinase inhibitor (TKI) Sunitinib in the clinic. But the severe toxic side effects associated with Sunitinib limit its therapeutic index. To improve the therapeutic index of Sunitinib, a prodrug strategy was employed to modify Sunitinib. The inactive prodrug AST-003 can be converted to Sunitinib *in vitro* and *in vivo*. Compared with Sunitinib, AST-003 has unique biochemical, cellular and pharmacokinetic properties with improved tolerability in mice and yield higher efficacy in tumor xenograft models. This prodrug strategy may constitute a novel paradigm to improve the therapeutic index of Sunitinib and other TKI or anti-angiogenesis drugs in general.

## Introduction

Tumor angiogenesis, which generates new blood vessels in a tumor, allows nutrients to be supplied to tumor cells to support their rapid growth. The essential role of angiogenesis in tumor growth and progression has been established in the laboratory and clinic [1], and many molecules have been implicated in this process [2]. Among them, vascular endothelial growth factor (VEGF) and its receptors have already been targeted in the clinic for their critical role in angiogenesis [3–5]. However, the therapeutic indices of angiogenesis inhibitors are generally limited [2,6–8].

The multi-targeted tyrosine kinase inhibitor (TKI) Sunitinib inhibits a variety of receptor tyrosine kinases (RTKs), including VEGFRs, Platelet-derived growth factor receptors (PDGFRs), c-kit and others, and exhibits potent anti-angiogenesis and anti-tumor growth activities [9–13]. Sunitinib has been approved by FDA in 2006 for the treatment of renal cell carcinoma (RCC) and Imatinib-resistant gastrointestinal stromal tumors (GISTs) [14,15]. However, the therapeutic index of Sunitinib in patients is very limited because Sunitinib treatment is associated with severe side effects, such as cardio-toxicity, hypothyroidism, hypertension, hematological toxicities, gastrointestinal disturbances, etc. [16,17], which are likely due to the high tissue distribution of Sunitinib [18].

Strategy to improve the therapeutic index of kinase inhibitors commonly relies on increasing the specificity and/or potency of these inhibitors. This strategy has been proven effective for many targeted therapies; however, applying this strategy to multi-targeted tyrosine kinase inhibitors is very challenging because the exact mechanism underlying their clinical efficacy is more complex than single-targeted kinase inhibitors. For example, the efficacy of Sunitinib is largely driven by its plasma concentration and angiogenesis inhibition [10], however, other properties of Sunitinib, such as the direct killing of cancer cells, also contribute to its clinical efficacy [19]. Furthermore, increasing only the potency of anti-VEGFR therapies has not effectively improved their therapeutic indices.

In this study, we aimed to improve the therapeutic index of Sunitinib by decreasing its high tissue distribution, which may be the cause of its high toxicity [18], while maintaining its anti-angiogenesis and anti-tumor function. We used an esterase-based prodrug strategy to modify Sunitinib without affecting its function as a multi-targeted tyrosine kinase inhibitor(TKI). The inactive prodrug AST-003 can be hydrolyzed to Sunitinib to exert biological functions. The pharmacokinetic profile of AST-003 differs from that of Sunitinib in mice. Compared with Sunitinib, AST-003 is better tolerated and associated with decreased toxicities in mice. In the tumor xenograft models, AST-003 can decrease tumor progression more efficiently than Sunitinib. The prodrug approach provides a novel strategy to improve the therapeutic indices of Sunitinib and other anti-angiogenesis inhibitors.

## Materials and Methods

### Compound preparation

Sunitinib was purchased from Huawen Chemical Company, Zhenzou, China. Compound AST-004 was synthesized as following: formaldehyde (30%, 405 mg, 5 mmol) and Et3N (506 mg, 5 mmol) were added to a solution of Sunitinib (0.5 g, 1.25 mmol) in DMF (14 ml) at 0°C. The mixture was stirred at room temperature overnight. After being cooled again to 0°C, H_2_O was added to the mixture while it was stirred. The resultant yellow precipitate was collected by filtration and dried in air to yield hydroxyl-methyl-Sunitinib (AST-004).

To synthesize AST-001, AST-004 (437 mg, 1.02 mmol) was dissolved in 5 ml of anhydrous DMF and 2 ml of pyridine. N, N-dimethylaminopyridine (12 mg, 0.1 mmol), N,N-dimethylglycine (206 mg, mmol) and EDC (384 mg, 2 mmol) were then added. The mixture was stirred at room temperature for 2 h. The solvent was removed under reduced pressure, and the product was extracted with DCM, washed with brine, dried over Na_2_SO_4_, and purified by column chromatography on silica gel using DCM/MeOH (0.1% Et3N was added to DCM). After the pure fractions were collected and dried under a vacuum, 50 mg of orange solid was obtained.

To prepare compounds AST-002 and AST-003, DMAP (50 mg) and either pivaloyl anhydride (2.87 g, 15.42 mmol) (AST-002) or (PhCO)_2_O (1.7 g, 7.7 mmol) (AST-003) were added to a solution of compound AST-004 (1.65 g, 3.86 mmol) in pyridine (50 ml). The mixture was stirred at room temperature overnight. After drying, saturated NaHCO_3_ solution (20 ml) was added, and the solution was extracted twice with EtOAc (20 ml each). The combined organic phase was dried (anhydrous Na_2_SO_4_), filtered, concentrated and purified by silica gel chromatography (eluting with DCM:MeOH = 10:1) to yield compound AST-002 or AST-003 as a yellow solid.

For *in vitro* assays, AST-002, AST-003 and Sunitinib were dissolved in DMSO to prepare 10 mM stock solutions and stored at -20°C. The AST-004 solution was freshly prepared for each experiment. For *in vivo* assays, AST-002, AST-003 and Sunitinib were dissolved in DMSO and Tween-80 (DMSO:Tween-80 = 1:1) and then diluted with PBS (pH 7.4) to reach final concentrations of DMSO and Tween-80 of 5%.

### Prodrug stability in plasma

The indicated compounds (10 μM) were added to mouse plasma with or without the esterase inhibitor paraoxon (3.75 μM). Samples were withdrawn in duplicate after 0, 5, 15, 30, 60 and 120 min at 37°C. Three volumes of cold methanol were then added to the samples to stop the reaction. After centrifugation at 15,000 rpm for 5 min to precipitate the protein, a 100-μl aliquot of the supernatant was removed for LC-MS/MS analysis.

### Cell lines and animals

The human non-small cell lung cancer cell lines A549 and NCI-H460, the human renal carcinoma cell lines 786-O and Caki-1, the human colon cancer cell line HT-29, and the human breast cancer cell line MDA-MB-231 were purchased from the Type Culture Collection of the Chinese Academy of Sciences in Shanghai, China. The human hepatocellular carcinoma cell line PLC/PRF/5 and murine hepatocellular carcinoma cell line Hepa1-6 were gifts from Dr. Lijian Hui at the Institute of Biochemistry and Cell Biology of the Shanghai Institutes for Biological Sciences (SIBCB), which were purchased from Type Culture Collection of the Chinese Academy of Sciences in Shanghai, China. The human hepatocellular carcinoma cell lines HUH-7, 7721, 7703, Bel-7402, and Sk-hep-1 were obtained from Dr. Jiang Li at the Sun Yat-Sen University School of Medicine, which were purchased from Type Culture Collection of the Chinese Academy of Sciences in Shanghai, China. HUVEC cells were purchased from Allcells, Inc. The cell lines A549, Caki-1, HT-29, MDA-MB-231, PLC/PRF/5, HUH-7, 7721, 7703, Bel-7402, Sk-hep-1 and Hepa1-6 were cultured in DMEM medium (Gibco, Life Technologies, China) containing 10% heat-inactivated (HI) fetal bovine serum (Gibco, Life Technologies, USA) and 1% Penicillin/Streptomycin (Hyclone), while the NCI-H460, 786-O and B16-F10 cell lines were maintained in RPMI-1640 medium (Gibco, Life Technologies, China) containing 10% HI fetal bovine serum (Gibco, Life Technologies, USA) and 1% Penicillin/Streptomycin (Hyclone).

All animals were obtained from the Laboratory Animal Center of Sun Yat-sen University. All animal experiments were carried out with the approval of the Ethical Committee of Sun Yat-Sen University. The animals were maintained under pathogen-free conditions and provided sterile food and water in accordance with the Guide for the Care and Use of Laboratory Animals of Guangdong Province. In the event of extreme stress and sickness, the animals were euthanized with the approval of the Ethical Committee of Sun Yat-Sen University.

### Compound treatment and western blots

To detect the changes in signaling proteins due to compound treatment, cancer cells were plated on 12-well plates at 2.5×10^5^ cells/well and incubated with 1 ml of medium plus 10% FBS at 37°C in 5% CO_2_ overnight. The cells were then washed twice with PBS and incubated in 1 ml of serum-free medium supplemented with 0.1% BSA (Amresco) with the indicated concentrations of compounds for 2 h. The cells were then washed once with 1 ml of cold PBS and lysed directly in 200 μl of lysis buffer containing a protease inhibitor cocktail (Roche). Fifty microliters of SDS loading buffer (5×) was added. The samples were then boiled at 95°C for 5 min and subjected to an SDS-PAGE and western blot analysis. The western blots were developed with chemiluminescent HRP substrate (Millipore Corporation, Billerica, USA) and detected using a Bio-Rad imager (ChemiDoc^TM^ XRS+).

All antibodies used in this study were from Cell Signalling, p-Stat3 (Y705) (D3A7) Rb mAb, Stat3 (79D7) Rb mAb, p-Akt (Ser473) mAb, Akt antibody, p-MEK1/2 (S217/221) (41G9) Rb mAb, MEK1/2 (47E6) Rb mAb, p-P44/42 MAPK (Thr202/Tyr204) mAb, p44/42 MAPK (Erk1/2) (137F5) Rb mAb, and GADPH (14C10) Rb mAb.

### *In vitro* cytotoxicity assays

Indicated cells were plated in 96-well plates with 50 μl of complete medium at 2,000 cells/well. After incubation overnight at 37°C, 5% CO_2_, 50 μl of complete medium with different concentrations of drugs was then added and incubated at 37°C in 5% CO_2_. At the indicated time, 10 μl of Cell Counting Kit-8 reagent (Dojindo) was added to each well. After incubation at 37°C in 5% CO_2_ for 1–4 h, the OD_450 nm_ was measured using a TECAN microplate reader (Infinite F50). The cytotoxic activities were calculated based on the OD_450 nm_ readings.

### Maximum tolerated dose (MTD) assays

Female BALB/c mice (>17 g) approximately 4~5 weeks old were used for the MTD assays. The animals were divided randomly into groups of 5 animals and were administered different concentrations of the compounds or vehicles. The weight of each mouse was measured and recorded daily. The gross health of each mouse was also observed and recorded. When an animal has weight loss over 25%, or is unable to move, or appears suffering, the animal is euthanized in accordance with the Guide for the Care and Use of Laboratory Animals of Guangdong Province. Upon the event of euthanization or unexpected death, histology and blood samples were analyzed to determine the cause of death. At the end of the experiment, serum samples and tissues were collected for further analyses.

### *In vivo* efficacy assays

A549 cells were harvested at 70~80% confluency and re-suspended in PBS (pH 7.4) to a final concentration of 2.5×10^7^cells/ml. Two hundred microliters (approximately 5×10^6^ cells) of the tumor cell suspension was injected subcutaneously into the right flanks of 4~5 week old female BALB/c-nu mice. The treatment was initiated when the tumor volumes reached approximately 75~200 mm^3^. The tumor volume was measured and calculated based on the formula *V* = length × (width)^2^/2. At the end of the experiments, blood, tissue, and tumor samples were collected for further analyses.

### Compound pharmacokinetic and tissue distribution studies

Animals (BALB/c mice were used unless indicated otherwise) were administered with compounds or vehicles via intraperitoneal (*i*.*p*.) or oral gavage (*p*.*o*.) delivery routes. At the indicated time, serum samples were collected for further analyses. For the tissue distribution studies, animals were sacrificed, and the tissue and plasma samples were then collected. After the tissues were weighed, 10x PBS was added to homogenize the samples. The amount of compounds and metabolites in the plasma or tissue samples was then determined by LC-MS.

## Results

### Sunitinib prodrugs can be hydrolyzed by esterase

**Fig 1:**
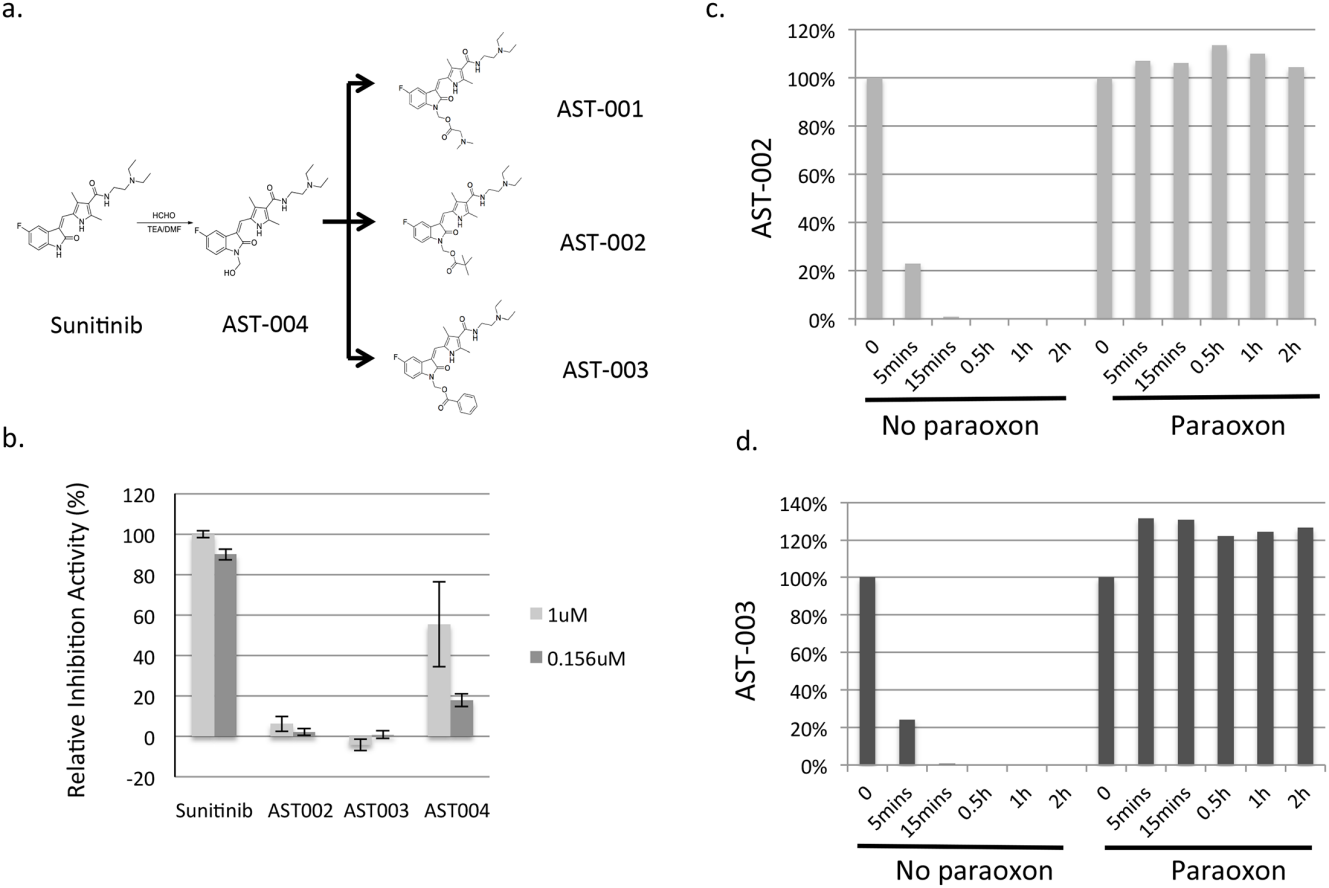
Synthesis and characterization of the prodrugs AST-001, AST-002, and AST-003. **(a)**, AST-001, AST-002 and AST-003 were synthesized as described in the Methods and Materials. **(b)**, The VEGFR2 biochemical assay was performed as described before using the recombinant VEGFR2 protein [21]. **(c)** and **(d)**, AST-002 and AST-003 were rapidly hydrolyzed to Sunitinib and AST-004 in serum (Y axis, the percentage of AST-002 or AST-003 still in the solution, 100% at time 0; X axis, incubation time). The *in vitro* plasma stability assays were performed as described in the Materials and Methods. The data represent the average of duplicate experiments.

We used an esterase-based prodrug strategy to modify Sunitinib. Based on the co-crystal structure of tyrosine kinase and Sunitinib, the pyrrole ring of Sunitinib is important for its ability to bind to the RTK catalytic domain [20]. We hypothesized that modifying the pyrrole group would disrupt the RTK inhibition activity of Sunitinib. After being hydrolyzed by esterase, Sunitinib would then be released and be biologically active similar to Sunitinib. AST-001 was synthesized based on this strategy (Fig 1a). AST-001 inhibited cancer cell growth *in vitro* with a potency similar to that of Sunitinib (S1 Table). More importantly, AST-001 administration has lower Sunitinib concentrations in multiple organs than Sunitinib administration (S1 Fig), which may reduce its toxicity; however, AST-001 was very unstable and rapidly hydrolyzed (data not shown). Therefore, more stable compounds, AST-002 and AST-003, were synthesized (Fig 1a).

Recombinant VEGFR2 was used to measure the ability of AST-002 and AST-003 to inhibit RTKs. AST-002 and AST-003 did not inhibit VEGFR2 activity (Fig 1b). The hydrolyzed intermediate AST-004 minimally inhibited VEGFR2 activity at 0.156 μM (Fig 1b). The low inhibitory activity of AST-004 was likely due to its hydrolysis to Sunitinib during assay incubation because AST-004 is unstable in salt solutions.

*In vitro* plasma testing showed that both AST-002 and AST-003 can be hydrolyzed to Sunitinib and compound AST-004 within 30 min (Fig 1c and 1d). The esterase inhibitor paraoxon can completely block the hydrolysis, suggesting that AST-002 and AST-003 can be efficiently hydrolyzed by esterase *in vivo*.

### Prodrugs inhibit RTK activity and tumor cell proliferation

**Fig 2:**
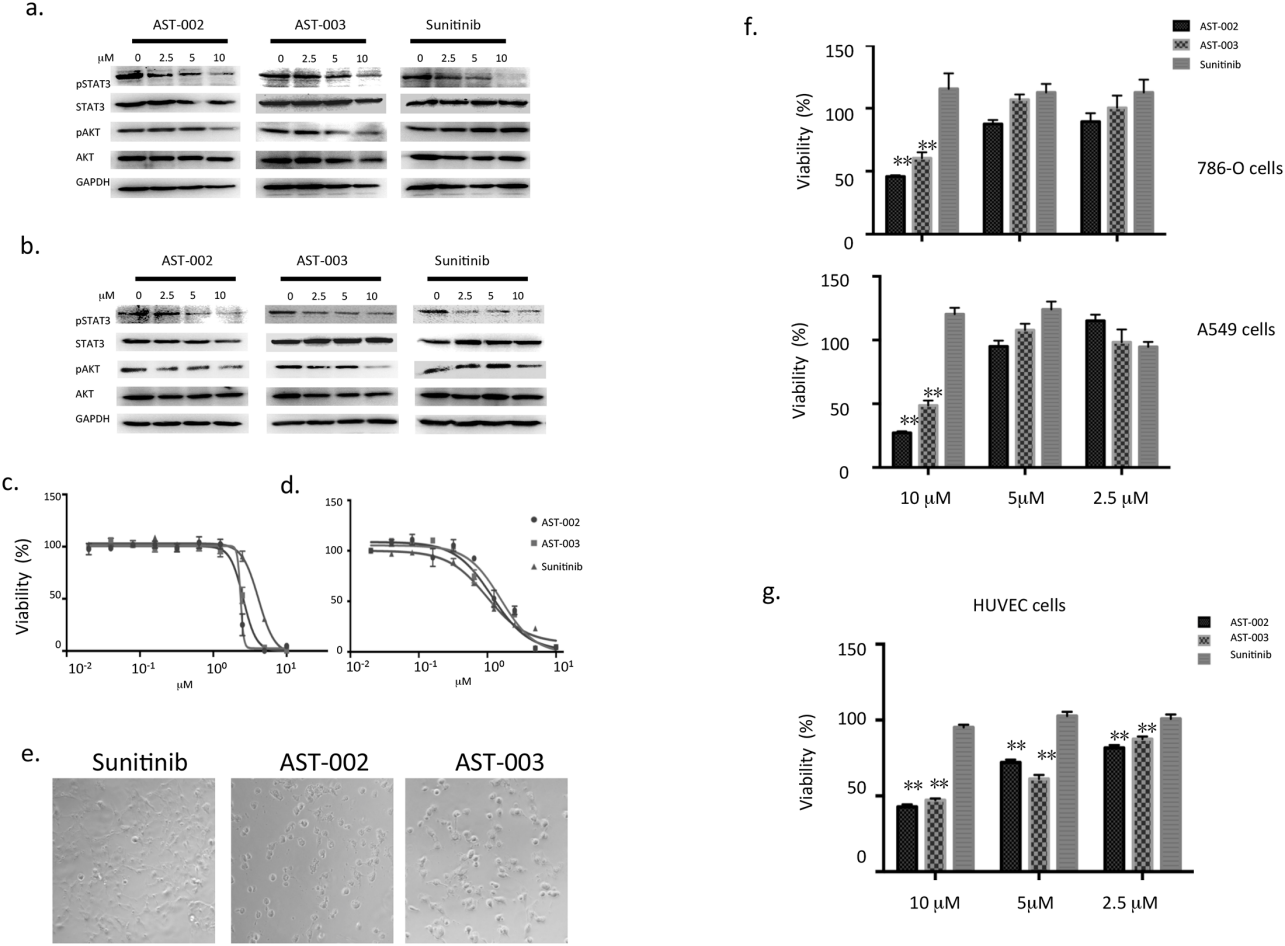
AST-002, AST-003, and Sunitinib induced the dephosphorylation of STA3 and AKT as well as cellular changes. 786-O cells **(a)** and A549 cells **(b)** were treated with different compounds at the indicated concentrations. After a 2-h treatment, the cells were collected for a western blot analysis to detect the levels of phospho-STAT3 and phospho-AKT. Cytotoxic assays were performed in 786-O cells **(c)** and A549 cells **(d)** as described in the Materials and Methods. Error bars are the standard deviations of three replicates. **(e)** The morphology of 786-O cells treated with the compounds for 4 h at 20×magnification. A549 and 786-O cells **(f)** and HUVEC cells **(g)** were treated with different compounds for 8 h, and the compounds were then removed. The cells were incubated in complete medium without compounds for another 16hrs. Cytotoxicity assays were then performed. All data are the means of 4 replicates, with error bars representing the standard deviation (** P<0.01 *vs*. Sunitinib).

Sunitinib has been shown to decrease the phosphorylation of a number of downstream signal proteins, including STAT3, MEK, ERK, and AKT [22–24]. To determine whether AST-002 and AST-003 function the same as Sunitinib in tumor cells, 786-O cells were treated with Sunitinib, AST-002, and AST-003. Similar to Sunitinib, both AST-002 and AST-003 decreased the phosphorylation of STAT3 and AKT (Fig 2a). Similar effects were also observed in the A549 cell line (Fig 2b). These results suggest that AST-002 and AST-003 can be hydrolyzed and inhibit signaling pathways in tumor cells.

To measure cancer cell growth inhibition, 786-O and A549 cells were treated with Sunitinib, AST-002 or AST-003. The IC50 values of all three compounds were similar for the two aforementioned cancer cell lines (Fig 2c and 2d), as well as 12 additional cancer cell lines (S2a Fig and S2 Table). These data further support the notion that AST-002 or AST-003 can be converted to Sunitinib to inhibit downstream signaling pathways and kill broad spectrum of tumor cells.

Different from Sunitinib, we observed that treatment with AST-002 or AST-003 caused rapid changes in cell morphology during incubation. Cancer cells appeared round and stressed as early as 4 h after incubation with AST-002 and AST-003, while the cells that were incubated with Sunitinib remained attached, elongated, and exhibited a normal healthy cell morphology (Fig 2e). An 8-h exposure to AST-002 or AST-003 led to significant cell death, while Sunitinib treatment for 8 h did not affect cell viability (Fig 2f), suggesting that AST-002 and AST-003 kill cancer cells more rapidly than Sunitinib; and this cytotoxic effect could not be reversed by removing AST-002 or AST-003 (Fig 2f). Though unstable in solution, AST-004 has similar rapid cancer cell killing to AST-002 and AST-003 (S2b Fig).

Because the major target of Sunitinib *in vivo* is the tumor vessel endothelial cell, the cytotoxic activities of AST-002 and AST-003 were also measured using human umbilical vein endothelial cells (HUVECs). Similar to 786-O and A549 cells, less than 50% of HUVECs were viable after an 8-h treatment with AST-002 or AST-003 (Fig 2g). Similarly rapid rates of killing were also observed for 24-h and 48-h incubations (S2c-S2e Fig). These data suggest that AST-002 and AST-003 likely penetrate and kill cancer cells and endothelial cells at a faster rate than Sunitinib.

### The toxicities of AST-003 are less than those of Sunitinib in mice

**Fig 3:**
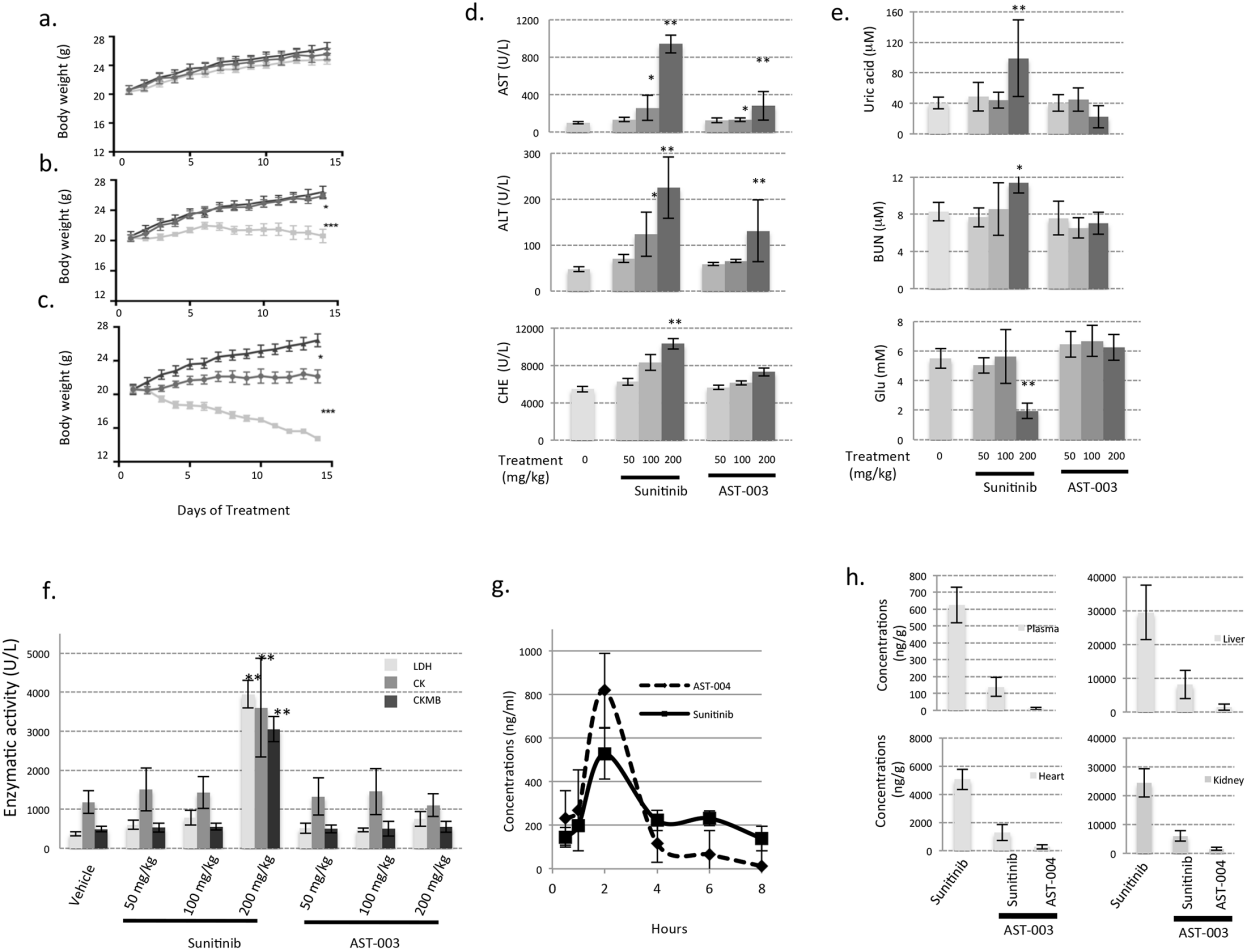
AST-003 is better tolerated in mice. The MTD experiments were performed as described in the Methods and Materials. AST-003 or Sunitinib was orally administered once daily at a dose of 50 mg/kg **(a)**, 100 mg/kg **(b)**, and 200 mg/kg **(c)**. The body weights of mice treated with AST-003 (circle), Sunitinib (square) and vehicle (triangle) were measured. All data are the means of 5 mice, with error bars representing the standard deviation. (Statistical analysis: **(b)** AST-003 *vs*. Sunitinib, *** P<0.001; Sunitinib *vs*. Vehicle, *** P<0.001; **(c)** AST-003 *vs*. Vehicle, * P<0.05; AST-003 *vs*. Sunitinib, *** P<0.001; Sunitinib *vs*. Vehicle, *** P<0.001) At the end of the MTD assays, the biochemical activities of AST, ALT, and CHE **(d)**, Glucose(Glu), Urea acid, blood urea nitrogen (BUN) **(e)**, LDH, CK, and CKMB **(f)** were measured (* P<0.05; ** P<0.01). **(g)** AST-003 was orally administered at a dose of 40 mg/kg. The concentrations of AST-004 and Sunitinib were then measured. All data are the means of replicates from 5 mice, with error bars representing the standard deviation. **(h)** AST-003 and Sunitinib were orally administered at a dose of 40 mg/kg. The concentrations of AST-004 and Sunitinib were then measured 8 h after administration. All data are the means of replicates from 5 mice, with error bars representing the standard deviation.

Sunitinib treatment leads to a number of side effects in patients. To determine whether AST-003 could reduce the toxicity associated with Sunitinib, the gross toxicities of Sunitinib and AST-003 were evaluated. Mice were orally administered 3 different doses (50 mg/kg, 100 mg/kg, 200 mg/kg) of Sunitinib and AST-003 once daily. At the dose of 50 mg/kg (Fig 3a), neither Sunitinib nor AST-003 treatments caused weight loss or apparent health problems. At the dose of 100 mg/kg (Fig 3b), mice treated with compound AST-003 exhibited normal weight gain and no apparent health problems; however, mice treated with Sunitinib did not gain weight and exhibited decreased movement. At the dose of 200 mg/kg (Fig 3c), mice treated with Sunitinib lost an average of over 20% of their weight, while mice treated with AST-003 maintained their weight. Furthermore, mice in the 200 mg/kg Sunitinib treatment group did not exhibit active movement and appeared sick upon gross examination at the end of experiments, while mice in the AST-003 200 mg/kg treatment group behaved similar to the control group. Similar to AST-003, AST-002 was also better tolerated than Sunitinib (S3a Fig), though less tolerated than AST-003. Similar results were also observed when compounds administrated intraperitoneally (data not shown). The body weight change caused by AST-003 was also reversible. Mice treated with 175 mg/kg AST-003 resumed growth immediately after the treatment was stopped, while growth remained stunted in the Sunitinib treatment group (S3b Fig).

To further analyze the toxicities, at the end of the 14-d treatment, blood samples were collected, and vital biochemical indicators were measured. In mice treated with 200 mg/kg Sunitinib, the levels of aspartate transaminase (AST), alanine transaminase (ALT), and cholinesterase (CHE) (Fig 3d) were significantly increased, indicating possible liver, heart, renal, or other tissue toxicities; the level of uric acid, blood urea nitrogen (Fig 3e) were higher, but glucose (Glu) was lower, indicating possible renal toxicity; the enzymatic activities of lactate dehydrogenase (LDH), creatine kinase (CK), creatine kinase-MB (CKMB) were also significantly higher (Fig 3f), indicating possible heart, and other organ toxicities. In contrast, except AST and ALT, the levels of these markers were in the normal range in mice treated with 200 mg/kg AST-003 (Fig 3f). These data further support the notion that AST-003 is less toxic than Sunitinib in mice, especially the liver heart, or renal toxicities.

Pharmacokinetic studies were performed to understand the decreased toxicity of AST-003. Because the major metabolites of AST-003 after incubation with mouse serum *in vitro* were AST-004 and Sunitinib (data not shown), the levels of both Sunitinib and AST-004 were measured *in vivo* after AST-003 administration. AST-004, which was very unstable as a pure compound *in vitro*, was detected as the majority of the derivatives of AST-003; however, it also disappeared more rapidly than Sunitinib in plasma (Fig 3g). In other tissues, including liver, heart, and kidney, the total levels of Sunitinib and AST-004 after AST-003 administration were also much lower than the same amount of Sunitinib administration 8 hours after administration (Fig 3h). Together, these data suggest that AST-003 is less toxic than Sunitinib because of the lower sustained level of Sunitinib and AST-004 after administration of AST-003.

### The efficacy of AST-003 is higher than that of Sunitinib in tumor xenograft models

**Fig 4:**
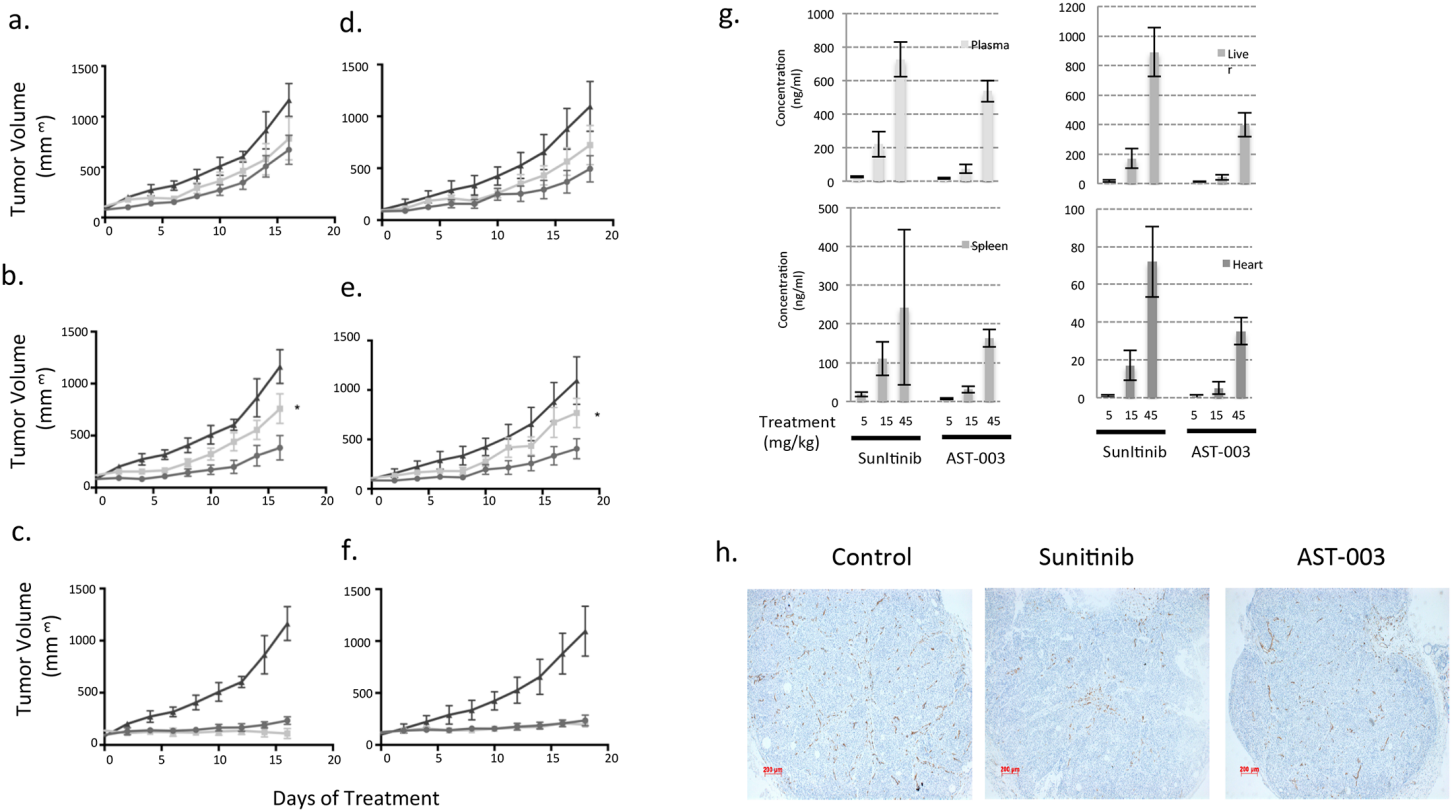
AST-003 more effectively inhibits tumors than Sunitinib. Mice transplanted with A549 cells were treated *i*.*p*. with AST-003 (circle), Sunitinib (square) or vehicle (triangle) at doses of 1.5 mg/kg **(a)**, 7.5 mg/kg **(b)**, and 30 mg/kg **(c)** (* AST-003 *vs*. Sunitinib, p<0.05). In a separate experiment, these drugs were orally administered at doses of 5 mg/kg **(d)**, 15 mg/kg **(e)**, and 45 mg/kg **(f)** (* AST-003 *vs*. Sunitinib, p<0.05). **(g)** The concentrations of Sunitinib in different tissues were measured after two weeks of compound treatment. All data are the means of replicates from 5 mice, with error bars representing the standard deviation. **(h)** Mice with established A549 xenografts were orally treated with at 15 mg/kg Sunitinib, AST-003, or vehicle once daily for two days. Tumor samples were retrieved and fixed using the Zinc method. CD31 staining was then performed. Representative figures from 6 independent fields are shown here.

While decreasing the level of Sunitinib can reduce its toxicity, a lower concentration of Sunitinib can also decrease its efficacy because the sustained level of Sunitinib is important for its anti-angiogenesis effect [10]. However, as a short exposure to AST-003 can reduce endothelial and cancer cell viability, AST-003 may still be able to inhibit tumor growth and lead to tumor growth inhibition. To test the efficacy of AST-003, A549 xenografted mice were treated with AST-003 or Sunitinib intraperitoneally. At the low dosage of 1.5 mg/kg daily, neither AST-003 nor Sunitinib significantly inhibited tumor growth (Fig 4a); however, at the high dose of 30 mg/kg (Fig 4c), both compounds strongly inhibited the tumors. At the dose of 7.5 mg/kg, AST-003 inhibited tumors significantly better than Sunitinib (Fig 4b), suggesting that AST-003 inhibits tumors more effectively *in vivo* than Sunitinib.

Oral dosing was also tested because Sunitinib is orally administered to patients in clinic. At the high dose of 45 mg/kg, both AST-003 and Sunitinib completely inhibited tumor growth (Fig 4f). At the lower doses (5 mg/kg and 15 mg/kg), AST-003 inhibited tumor growth better than Sunitinib (Fig 4d and 4e). Consistent with the acute pharmacokinetic studies (Fig 3), the levels of Sunitinib at the end of the 2-week treatment period were also lower in mice treated with AST-003 (Fig 4g), further confirming that sustained levels of Sunitinib in the tissues are not required for AST-003 efficacy.

As a potent angiogenesis inhibitor, Sunitinib disrupts the vascular structure in the tumor to kill tumor cells [10]. CD31 staining revealed that even following a 2-d treatment at the dose of 15 mg/kg, AST-003 and Sunitinib inhibited the formation of vascular structures (Fig 4h), suggesting that AST-003 is anti-angiogenic *in vivo*.

## Discussion

In this study, we aimed to improve the therapeutic index of Sunitinib using a prodrug strategy. The prodrug AST-003, which does not inhibit VEGFR kinase activity itself, can be hydrolyzed by esterase (Fig 1). In mice, after a transient increase in hydroxyl-methyl-Sunitinib AST-004 and Sunitinib, tissue exposure to AST-004 plus Sunitinib was lower for AST-003 treatment than for Sunitinib treatment (Fig 3). The decreased AST-004 plus Sunitinib level increased the tolerance, and reduced the toxicity of AST-003 comparing with Sunitinib (Fig 3). Furthermore, AST-003 exhibited much better efficacy than Sunitinib *in vivo* (Fig 4), likely due to its faster cell killing than Sunitinib (Fig 2). These unique properties of AST-003 suggest that AST-003 may have potential better therapeutic index than Sunitinib in cancer therapy.

Sunitinib is a multi-targeted tyrosine kinase inhibitor with potent anti-VEGFR activity. Although the efficacy of Sunitinib is largely driven by its angiogenesis inhibition [10], increasing the potency of its anti-VEGFR activity alone is likely not sufficient to improve the therapeutic index of Sunitinib as various potent VEGFR antagonists, including large and small molecules, exhibit limited efficacy [6–8], and highly selective small anti-VEGFR inhibitors have failed to prolong survival in the clinic thus far due to toxicity [2,19].

As a multi-targeted tyrosine kinase inhibitor, Sunitinib can directly inhibit cell proliferation and survival signaling in cancer cells, which also contributes to its clinical efficacy. However, the mechanism by which Sunitinib directly kills cancer cells is complex and not well understood except the direct inhibition of c-kit in GIST [19]. Because the clinical efficacy of Sunitinib is likely due to a combination of multiple kinase inhibitions [19], improving the inhibition potency of a subset of kinase(s) is unlikely to improve its anti-tumor effect in RCC.

To improve the therapeutic index of Sunitinib, reducing the toxicity of Sunitinib is very important. In this study, a prodrug strategy was used to modify Sunitinib because prodrug strategy has been successfully employed to improve drug solubility, tumor targeting, pharmacokinetics, and therefore overall therapeutic potency of various compounds, such as anti-viral drugs [25]. We focused on decreasing the tissue distribution of Sunitinib because the high tissue distribution of Sunitinib may be responsible for its high toxicity [18]. Our studies showed that the administration of AST-003 decreased the levels of Sunitinib in various tissues. Short-term maximum dosage testing showed that mice can tolerate at least two-fold increased doses of AST-003 and AST-002 than Sunitinib. The biochemical analysis also revealed that enzymes associated with cardiac, liver, and renal functions are less affected by AST-003 treatment than by Sunitinib treatment, suggesting overall decreased toxicity of AST-003.

Unexpectedly, the decreased tissue distribution of AST-003 did not decrease its tumor inhibition efficacy. AST-003 actually showed better efficacy in tumor xenograft models, probably due to its faster and irreversible cancer cell and endothelial cell killing by AST-003 or its metabolite AST-004 *in vivo* (Fig 2).

**Fig 5:**
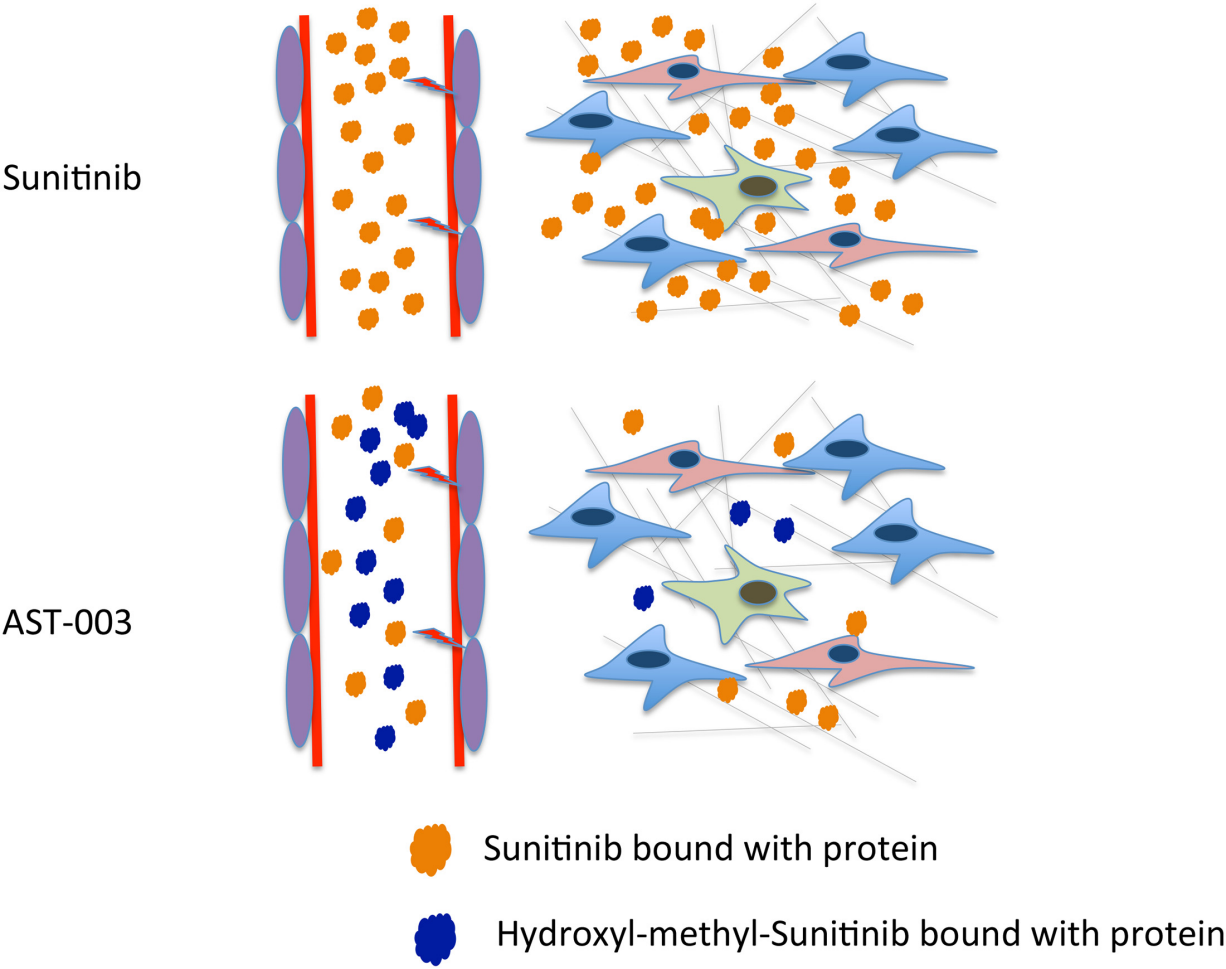
The proposed mechanism of AST-003. In mice treated with AST-003, the total Sunitinib (Sunitinib plus hydroxyl-methyl-Sunitinib) inhibits angiogenesis and cancer cell growth better than Sunitinib treatment alone because hydroxyl-methyl-Sunitinib kills vascular endothelial cells and cancer cells at a faster rate than Sunitinib. However, as hydroxyl-methyl-Sunitinib is less stable than Sunitinib, the lower sustained tissue concentrations of Sunitinib associated with AST-003 treatment are responsible for the observed reduced toxicity. Thus, AST-003 exhibits higher efficacy and lower systemic toxicity than Sunitinib.

Based on the experiments in this study, the following model was proposed (Fig 5). After AST-003 is absorbed *in vivo*, it is rapidly hydrolyzed into hydroxyl-methyl Sunitinib AST-004 and then Sunitinib. While hydroxyl-methyl Sunitinib AST-004 comprised the majority of the AST-003 derivatives, its levels were transient. Thus, the sustained concentrations of AST-004 and Sunitinib in the different tissues were low, which decreased the toxicity. The improved efficacy of AST-003 was likely due to the faster and irreversible killing activity of AST-003 and AST-004 on endothelial and cancer cells (Figs 2 and 4). Thus, the unique pharmacokinetics of AST-003, combined with its ability to rapidly kill cancer cells, resulted in the superior therapeutic efficacy of AST-003 *in vivo*.

Our data suggest that the therapeutic index of multi-targeted TKIs can be improved by changing their drug properties rather than kinase inhibition activities. Because improving the kinase inhibition activities of a highly potent multi-targeted TKI is very challenging, our strategy provides an alternative approach to developing the new generation of multi-targeted TKIs or angiogenesis inhibitors in general. Understandably, the therapeutic effects of AST-003 in humans have not yet been tested. Because anti-angiogenesis inhibitors can have potent anti-tumor activities in mice but much lower anti-tumor activities in human patients, the therapeutic effect of AST-003 need to be tested in patients.

More studies are needed to help understand the mechanisms of AST-003. For example, the kinetics of AST-003, AST-004 and Sunitinib in mice likely does not follow a simple linear conversion of AST-003 to AST-004 and then to Sunitinib. Because AST-004 is very unstable in solution, more studies are needed to understand how the levels of AST-003, AST-004 and Sunitinib are maintained *in vitro* and *in vivo*; and how they cooperatively affect cancer cell viability. Because Sunitinib can be metabolized to a variety of metabolites, further studies are necessary to identify possible metabolites of AST-003 and AST-004 other than Sunitinib and whether those metabolites will affect tumor inhibition and toxicity. These studies will improve our understanding of AST-003 and its potential benefits to patients.

## Supporting Information

S1 Fig Pharmacokinetic studies of Sunitinib (solid line) and AST-001 (dashed line) in mice. Pharmacokinetic studies are performed as described in the Materials and Methods using Kunmin mice. The concentration of Sunitinib in different tissues: a. Plasma; b. Heart; c, Liver; d, Spleen; e, Lung; f, Kidney. Each data point represents the average measurements from 6 mice. The error bars represent standard deviation.
(PPTX)

S2 Fig **(a)**, Cytotoxic assays of different cell lines. Cytotoxic assays were performed as described in Materials and Methods. 12 different cancer cell lines and HUVEC cells were treated with Sunitinib, AST-002 and AST-003. The data are shown as average of three independent replicates with error bars representing standard deviation. **(b)**, A549 cells were treated with different compounds for 8 hours, then cytotoxic assays were performed. All data are the means of 4 replicates with error bars representing standard deviation (** P<0.01 *vs*. Sunitinib). Note: for AST-004, compound needs to be freshly prepared as AST-004 is unstable in solution. 20% of AST-004 is already converted to Sunitinib within 5 mins adding to the medium (data not shown). 786-O **(c)** and A549 cells **(d)**, and HUVEC cells **(e)** were treated with different compounds for 24 hrs, then cytotoxic assays were performed. All data are the means of 4 replicates with error bars representing standard deviation (** P<0.01 *vs*. Sunitinib).
(PPTX)

S3 Fig **(a)**, MTD assays for AST-002 and AST-003. MTD assays were performed as described in the Materials and Methods. 4–5 weeks old female BALB/c mice (~20g) were administrated with different concentrations of compounds or vehicle. The weight of each mouse was measured and recorded daily. Two mice died in the Sunitinib 200mg/kg group at day 14 and day 16. **(b)**, AST-003 or Sunitinib were administered *p*.*o*. at dosage of 175mg/kg and interrupted between day 7 to day 9. AST-003 (circle), Sunitinib (square) and vehicle (triangle). (Statistical analysis: AST-003 *vs* Vehicle, * P<0.05; AST-003 *vs* Sunitinib, *** P<0.001; Sunitinib *vs* Vehicle, *** P<0.001).
(PPTX)

S1 Table IC50 of AST-001 cytotoxic assay. Cytotoxic assays were performed as described in Materials and Methods. 5 different tumor cell lines were treated with Sunitinib and AST-001. The data are shown as representative of three independent experiments.
(PPTX)

S2 Table IC50 of AST-002, AST-003, and Sunitinib. Cytotoxic assays were performed as described in Materials and Methods. The data are shown as representative of three independent experiments.
(PPTX)
