# Supplementary material for: Prodrug AST-003 Improves the Therapeutic Index of the Multi-Targeted Tyrosine Kinase Inhibitor Sunitinib
Source: PLoS One. 2015 Oct 29;10(10):e0141395. doi: 10.1371/journal.pone.0141395 (PMC4626378; doi:10.1371/journal.pone.0141395)
Supplement: S1 Fig — Pharmacokinetic studies are performed as described in the Materials and Methods using Kunmin mice. The concentration of Sunitinib in different tissues: a. Plasma; b. Heart; c, Liver; d, Spleen; e, Lung; f, Kidney. Each data point represents the average measurements from 6 mice. The error bars represent standard deviation. (PPTX) [file pone.0141395.s001.pptx]

## Slide 1
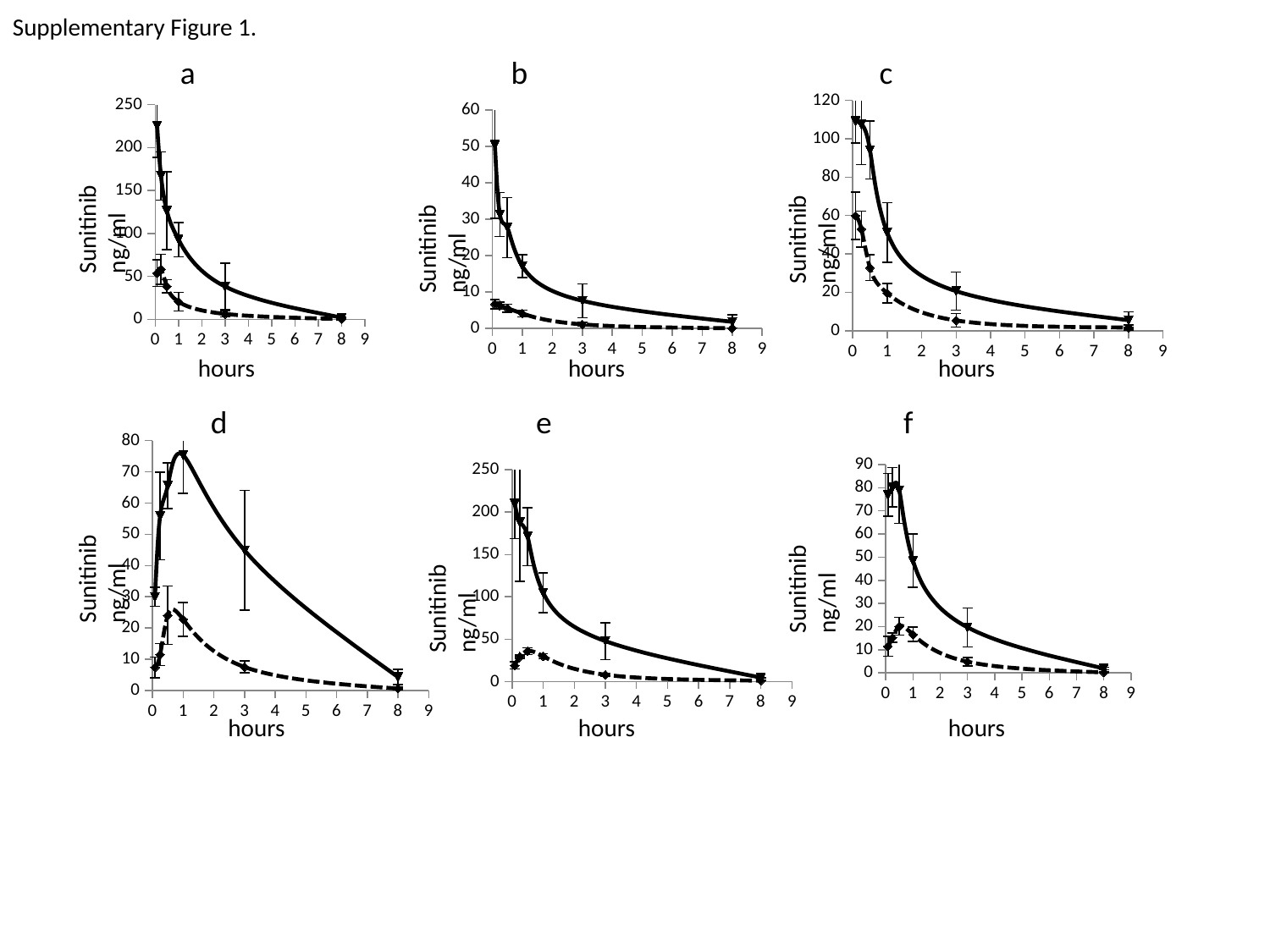

Supplementary Figure 1.
a b c
### Chart
| Category | A给药组 | S给药组 |
|---|---|---|
### Chart
| Category | A给药组 | S给药组 |
|---|---|---|
### Chart
| Category | A给药组 | S给药组 |
|---|---|---|Sunitinib ng/ml
Sunitinib ng/ml
Sunitinib ng/ml
hours
hours
hours
d e f
### Chart
| Category | A给药组 | S给药组 |
|---|---|---|
### Chart
| Category | A给药组 | S给药组 |
|---|---|---|
### Chart
| Category | A给药组 | S给药组 |
|---|---|---|Sunitinib ng/ml
Sunitinib ng/ml
Sunitinib ng/ml
hours
hours
hours
