# Supplementary material for: Prodrug AST-003 Improves the Therapeutic Index of the Multi-Targeted Tyrosine Kinase Inhibitor Sunitinib
Source: PLoS One. 2015 Oct 29;10(10):e0141395. doi: 10.1371/journal.pone.0141395 (PMC4626378; doi:10.1371/journal.pone.0141395)
Supplement: S2 Fig — (a), Cytotoxic assays of different cell lines. Cytotoxic assays were performed as described in Materials and Methods. 12 different cancer cell lines and HUVEC cells were treated with Sunitinib, AST-002 and AST-003. The data are shown as average of three independent replicates with error bars representing standard deviation. (b), A549 cells were treated with different compounds for 8 hours, then cytotoxic assays were performed. All data are the means of 4 replicates with error bars representing standard deviation (** P<0.01 vs. Sunitinib). Note: for AST-004, compound needs to be freshly prepared as AST-004 is unstable in solution. 20% of AST-004 is already converted to Sunitinib within 5 mins adding to the medium (data not shown). 786-O (c) and A549 cells (d), and HUVEC cells (e) were treated with different compounds for 24 hrs, then cytotoxic assays were performed. All data are the means of 4 replicates with error bars representing standard deviation (** P<0.01 vs. Sunitinib). (PPTX) [file pone.0141395.s002.pptx]

## Slide 1
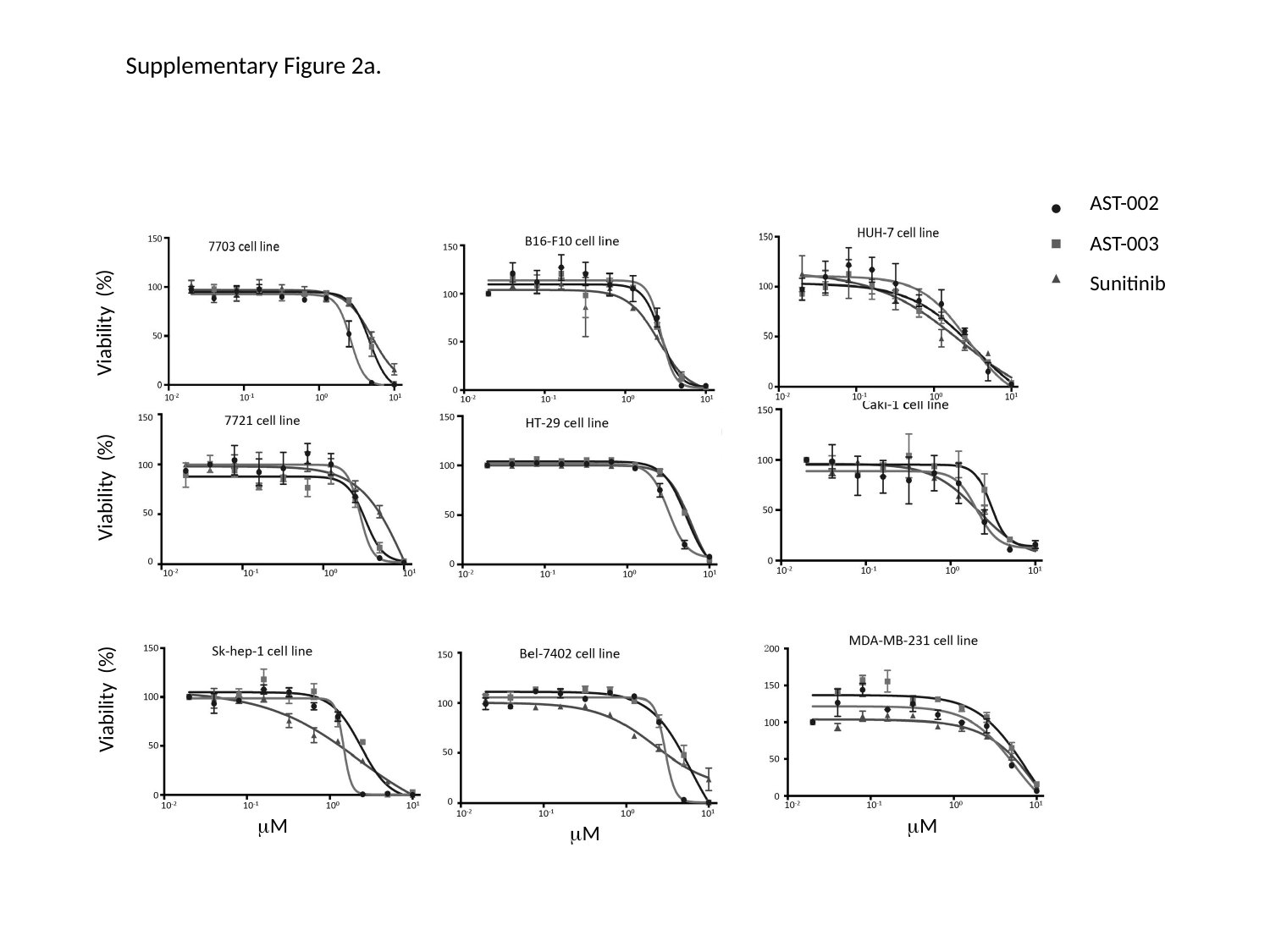

Supplementary Figure 2a.
AST-002
AST-003
Sunitinib
Viability (%)
Viability (%)
Viability (%)
mM
mM
mM

## Slide 2
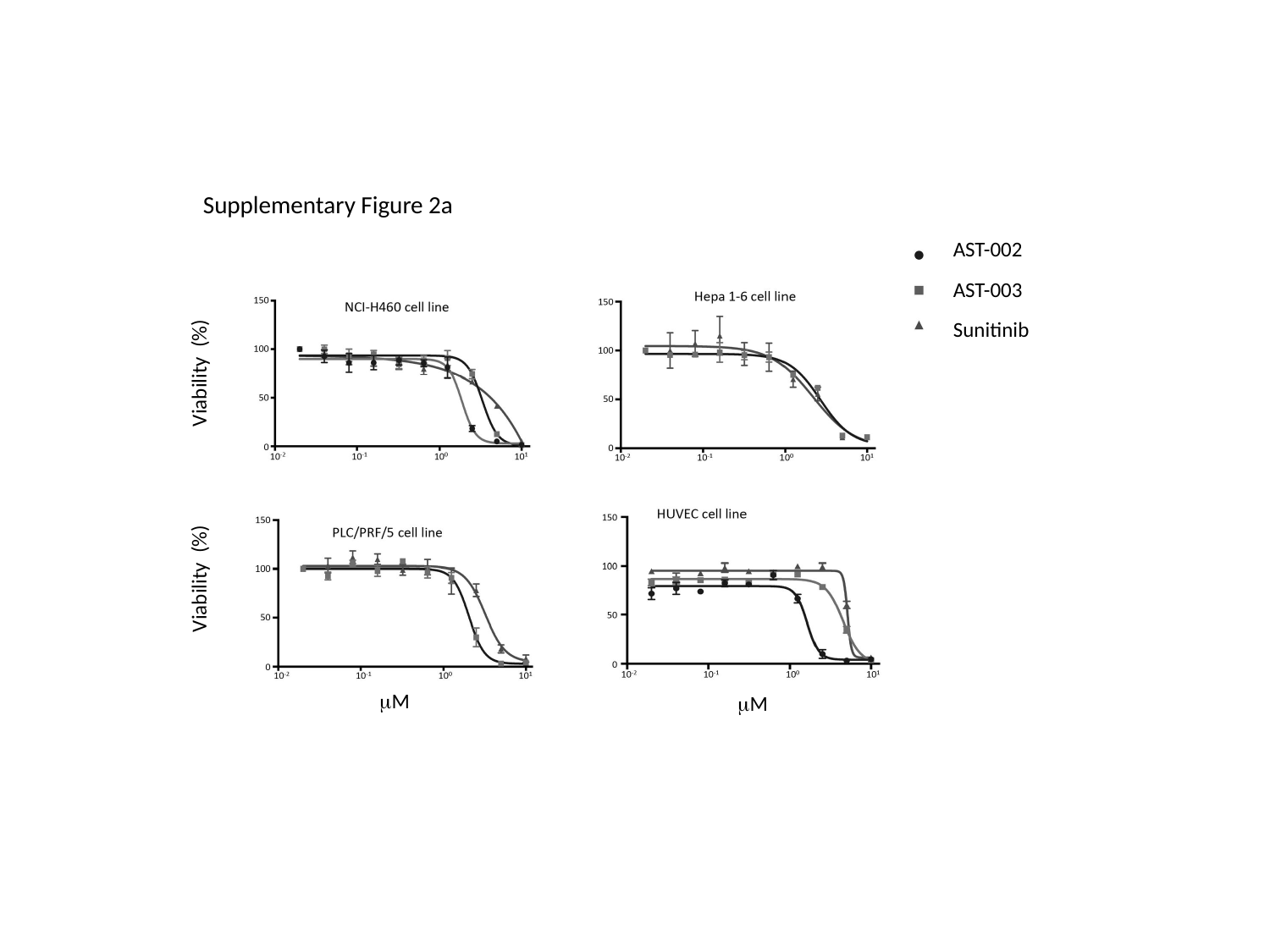

Supplementary Figure 2a
AST-002
AST-003
Sunitinib
Viability (%)
Viability (%)
mM
mM

## Slide 3
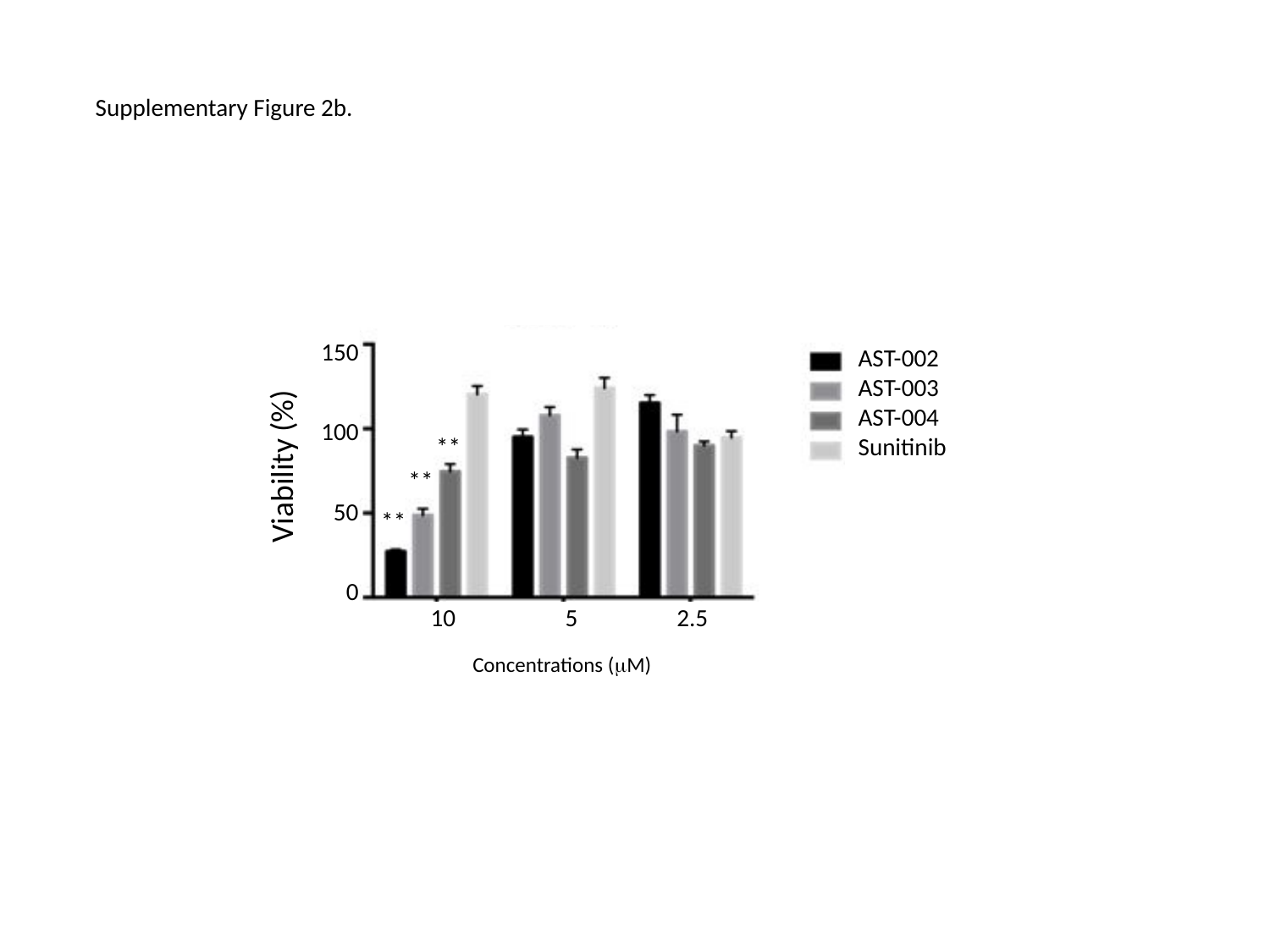

Supplementary Figure 2b.
150
100
50
0
AST-002
AST-003
AST-004
Sunitinib
**
Viability (%)
**
**
10 5 2.5
Concentrations (mM)

## Slide 4
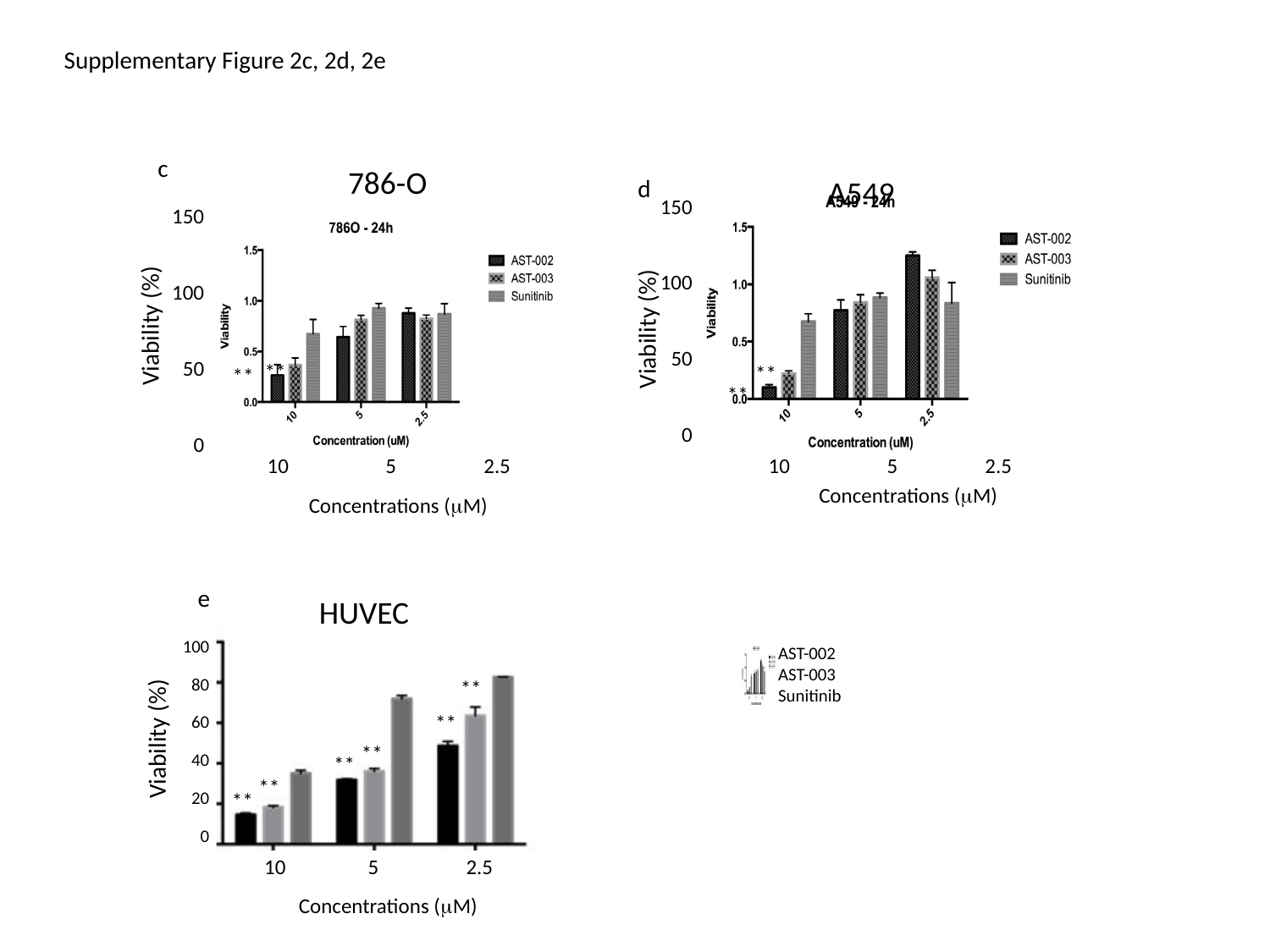

Supplementary Figure 2c, 2d, 2e
c
786-O
A549
d
150
100
50
0
150
100
50
0
Viability (%)
Viability (%)
**
**
**
**
10 5 2.5
10 5 2.5
Concentrations (mM)
Concentrations (mM)
e
HUVEC
100
80
60
40
20
0
AST-002
AST-003
Sunitinib
**
**
Viability (%)
**
**
**
**
10 5 2.5
Concentrations (mM)
