# Supplementary material for: Prodrug AST-003 Improves the Therapeutic Index of the Multi-Targeted Tyrosine Kinase Inhibitor Sunitinib
Source: PLoS One. 2015 Oct 29;10(10):e0141395. doi: 10.1371/journal.pone.0141395 (PMC4626378; doi:10.1371/journal.pone.0141395)
Supplement: S3 Fig — (a), MTD assays for AST-002 and AST-003. MTD assays were performed as described in the Materials and Methods. 4–5 weeks old female BALB/c mice (~20g) were administrated with different concentrations of compounds or vehicle. The weight of each mouse was measured and recorded daily. Two mice died in the Sunitinib 200mg/kg group at day 14 and day 16. (b), AST-003 or Sunitinib were administered p.o. at dosage of 175mg/kg and interrupted between day 7 to day 9. AST-003 (circle), Sunitinib (square) and vehicle (triangle). (Statistical analysis: AST-003 vs Vehicle, * P<0.05; AST-003 vs Sunitinib, *** P<0.001; Sunitinib vs Vehicle, *** P<0.001). (PPTX) [file pone.0141395.s003.pptx]

## Slide 1
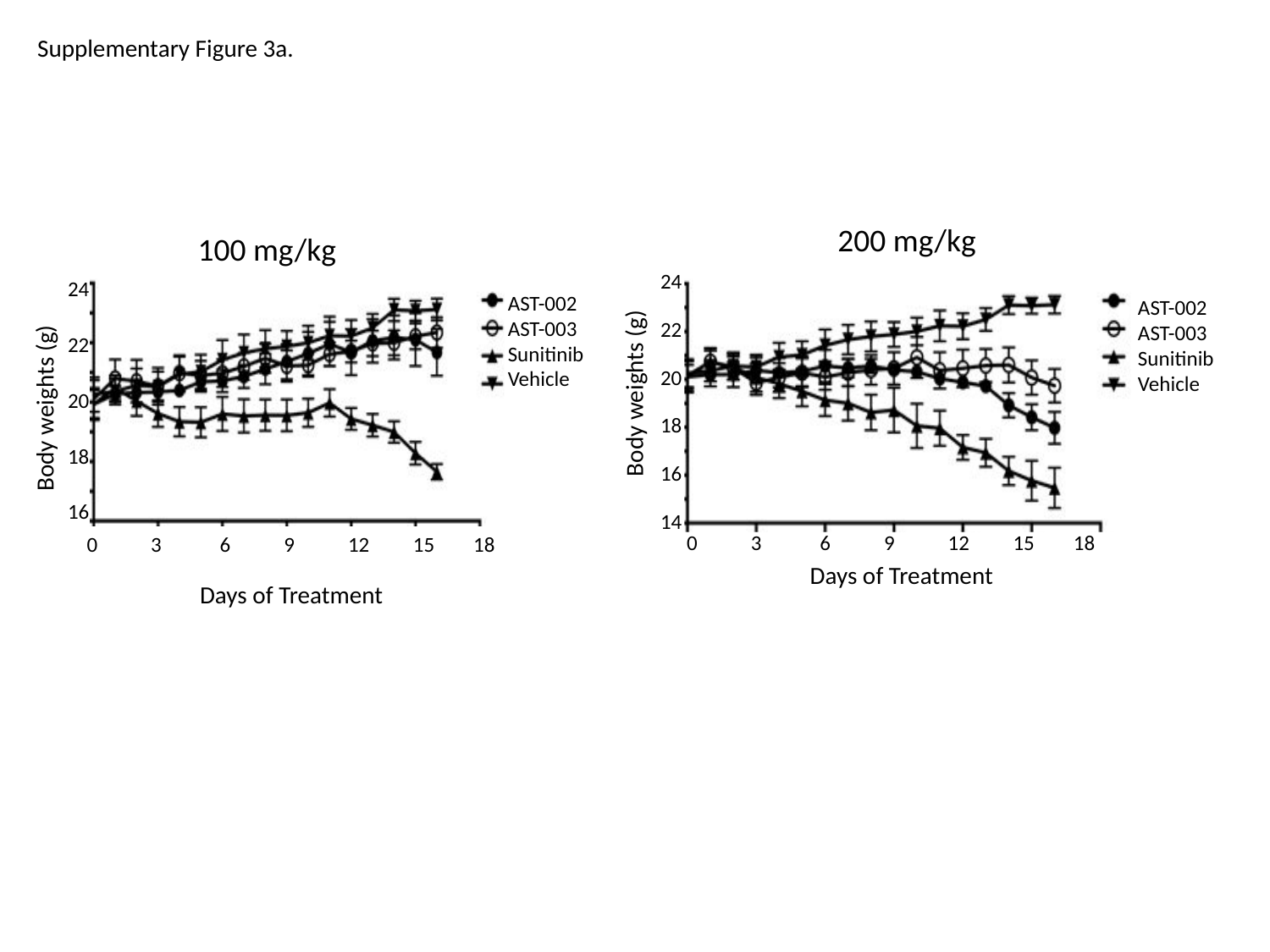

Supplementary Figure 3a.
200 mg/kg
100 mg/kg
24
22
20
18
16
14
24
22
20
18
16
AST-002
AST-003
Sunitinib
Vehicle
AST-002
AST-003
Sunitinib
Vehicle
Body weights (g)
Body weights (g)
0 3 6 9 12 15 18
0 3 6 9 12 15 18
Days of Treatment
Days of Treatment

## Slide 2
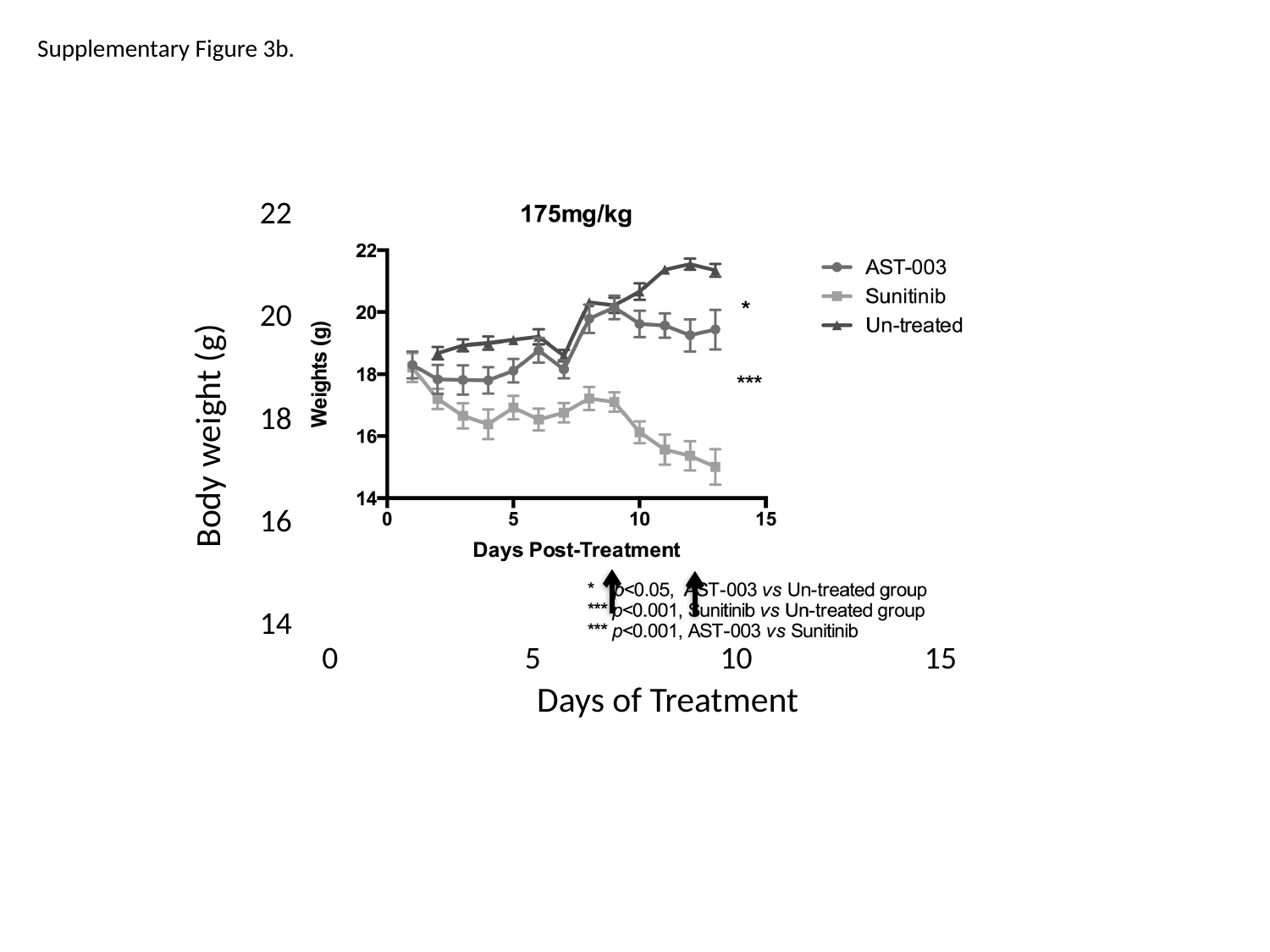

Supplementary Figure 3b.
22
20
18
16
14
Body weight (g)
0 5 10 15
Days of Treatment
