# Supplementary figures and images for: Prodrug AST-003 Improves the Therapeutic Index of the Multi-Targeted Tyrosine Kinase Inhibitor Sunitinib
Source: PLoS One. 2015 Oct 29;10(10):e0141395. doi: 10.1371/journal.pone.0141395 (PMC4626378; doi:10.1371/journal.pone.0141395)

## Slide 1
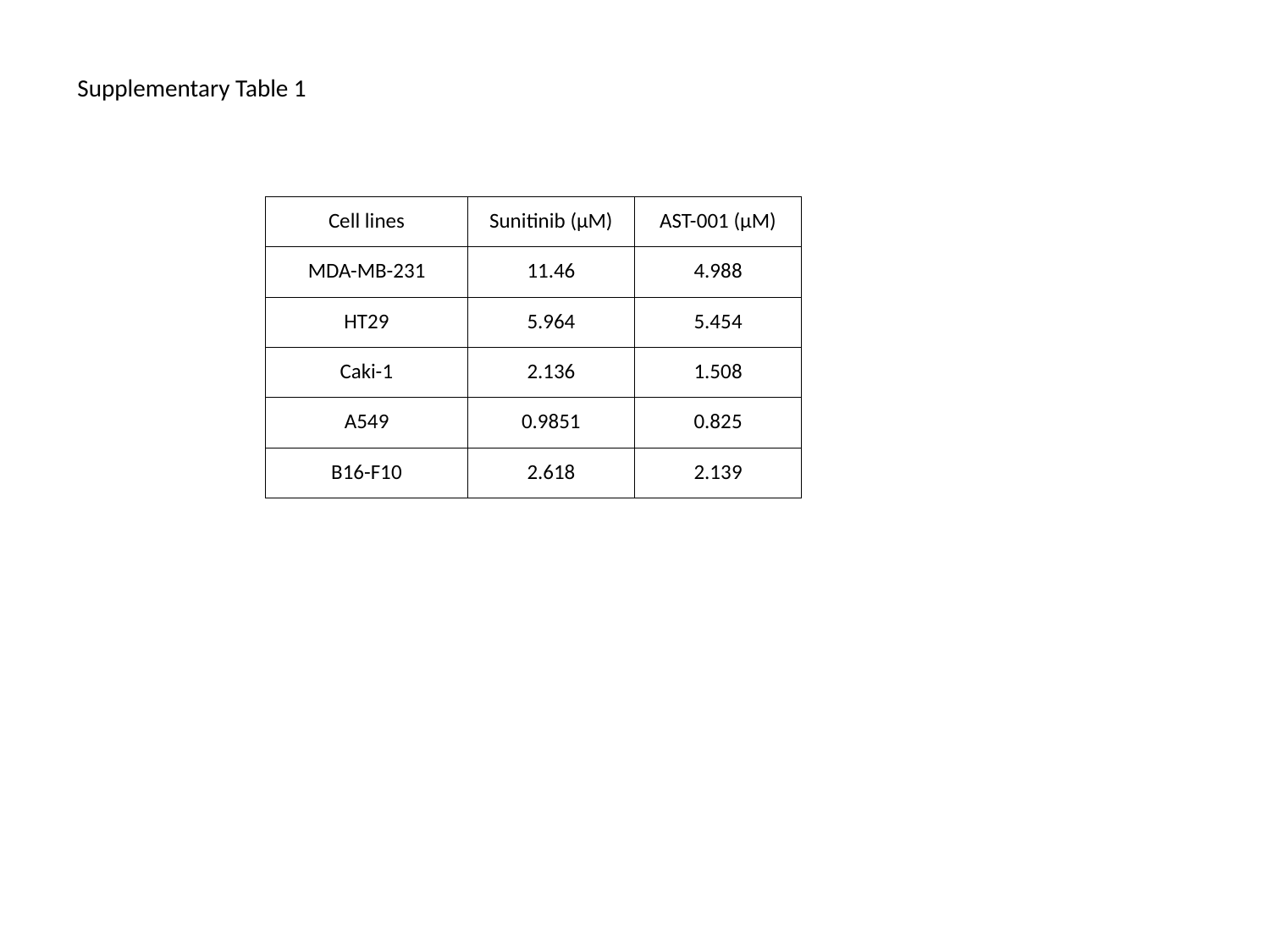

Supplementary Table 1
| Cell lines | Sunitinib (μM) | AST-001 (μM) |
| --- | --- | --- |
| MDA-MB-231 | 11.46 | 4.988 |
| HT29 | 5.964 | 5.454 |
| Caki-1 | 2.136 | 1.508 |
| A549 | 0.9851 | 0.825 |
| B16-F10 | 2.618 | 2.139 |

Supplement: S1 Table — Cytotoxic assays were performed as described in Materials and Methods. 5 different tumor cell lines were treated with Sunitinib and AST-001. The data are shown as representative of three independent experiments. (PPTX) [file pone.0141395.s004.pptx]
