# Supplementary material for: Prodrug AST-003 Improves the Therapeutic Index of the Multi-Targeted Tyrosine Kinase Inhibitor Sunitinib
Source: PLoS One. 2015 Oct 29;10(10):e0141395. doi: 10.1371/journal.pone.0141395 (PMC4626378; doi:10.1371/journal.pone.0141395)
Supplement: S2 Table — Cytotoxic assays were performed as described in Materials and Methods. The data are shown as representative of three independent experiments. (PPTX) [file pone.0141395.s005.pptx]

## Slide 1
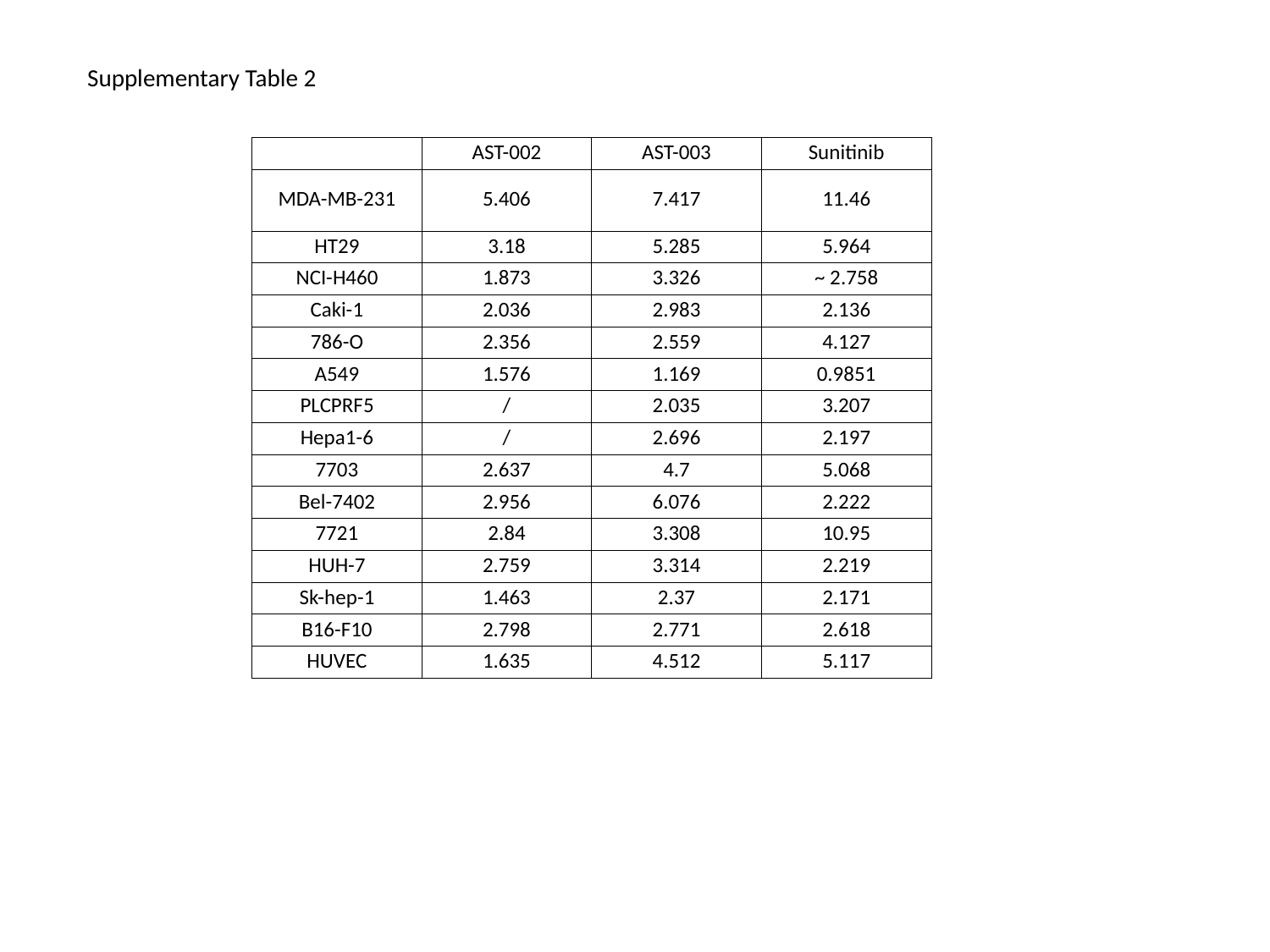

Supplementary Table 2
| | AST-002 | AST-003 | Sunitinib |
| --- | --- | --- | --- |
| MDA-MB-231 | 5.406 | 7.417 | 11.46 |
| HT29 | 3.18 | 5.285 | 5.964 |
| NCI-H460 | 1.873 | 3.326 | ~ 2.758 |
| Caki-1 | 2.036 | 2.983 | 2.136 |
| 786-O | 2.356 | 2.559 | 4.127 |
| A549 | 1.576 | 1.169 | 0.9851 |
| PLCPRF5 | / | 2.035 | 3.207 |
| Hepa1-6 | / | 2.696 | 2.197 |
| 7703 | 2.637 | 4.7 | 5.068 |
| Bel-7402 | 2.956 | 6.076 | 2.222 |
| 7721 | 2.84 | 3.308 | 10.95 |
| HUH-7 | 2.759 | 3.314 | 2.219 |
| Sk-hep-1 | 1.463 | 2.37 | 2.171 |
| B16-F10 | 2.798 | 2.771 | 2.618 |
| HUVEC | 1.635 | 4.512 | 5.117 |
